# UNDERSTANDING THE PALLIATIVE CARE INFORMATION CIRCULATING ON TWITTER DURING THE CORONAVIRUS PANDEMIC

**DOI:** 10.1093/geroni/igac059.759

**Published:** 2022-12-20

**Authors:** Megumi Inoue, Mahdi Hashemi, Meng-Hao Li, Rajendra Kulkarni, Naoru Koizumi

**Affiliations:** George Mason University, Fairfax, Virginia, United States; George Mason University, Fairfax, Virginia, United States; George Mason University, Fairfax, Virginia, United States; George Mason University, Fairfax, Virginia, United States; George Mason University, Fairfax, Virginia, United States

## Abstract

Palliative care is a growing medical specialty. While its value has become particularly evident during the pandemic, it faces various challenges including misconceptions among the general public, a lack of awareness of its benefits, and limited/sporadic access and coverage by Medicare. Given the increasing number of people sharing their health information and seeking healthcare information on social media where misinformation is widely spread, this study examined types of information on palliative care circulated on Twitter. A total of 26,495 English Tweets were collected 01/01/2020 – 12/30/2021 using the keywords “Palliative” and “Covid” or “Corona”. We manually developed a framework for coding/classifying the first 6,000 Tweets. Of those, 5,308 were unrelated Tweets (e.g., advertising palliative care seminars/conferences). Among the remaining Tweets, persistent myths were observed (e.g., palliative care is only for dying people) and were labeled accordingly. In addition, while some people mentioned negative impact of the pandemic on palliative care (e.g., shortage of beds), others found value in palliative care and reported positive changes due to the pandemic (e.g., telehealth in palliative care). Consequently, the following categories were defined for Tweets: i) Recognized benefits (203 Tweets); ii) Positive impact of the pandemic on palliative care (120 Tweets); iii) Negative impact of the pandemic on palliative care (333 Tweets); iv) Myth (63 Tweets). We will use these manually classified Tweets to train machine-learning algorithms to automatically classify the remaining tweets, aiming to obtain a comprehensive understanding of palliative care information circulating on Twitter and seek ways to promote palliative care use.

